# Antagomir of miR-31-5p modulates macrophage polarization via the AMPK/SIRT1/NLRP3 signaling pathway to protect against DSS-induced colitis in mice

**DOI:** 10.18632/aging.205651

**Published:** 2024-03-11

**Authors:** Yuyi Yuan, Shuangjiao Deng, Jia Yang, Zhexing Shou, Chunzhu Wei, Lijuan Zhang, Feng Zhu, Fei Gao, Xingxing Liu, Yujin Liu, Qianyun Chen, Heng Fan

**Affiliations:** 1 Department of Integrated Traditional Chinese and Western Medicine, Union Hospital, Tongji Medical College, Huazhong University of Science and Technology, Wuhan 430022, China

**Keywords:** miR-31-5p antagomir, ulcerative colitis, AMPK, macrophage, intestinal barrier dysfunction

## Abstract

Macrophage-driven immune dysfunction of the intestinal mucosa is involved in the pathophysiology of ulcerative colitis (UC). Emerging evidence indicates that there is an elevation in miR-31-5p levels in UC, which is accompanied by a downregulation of adenosine 5′-monophosphate (AMP)-activated protein kinase (AMPK) expression. Nevertheless, the precise influence of miR-31-5p on macrophage polarization and the integrity of the intestinal epithelial barrier in UC remains to be fully elucidated. This study explored the role of miR-31-5p and AMPK in UC through a bioinformatics investigation. It investigated the potential of miR-31-5p antagomir to shift macrophages from pro-inflammatory M1 phenotype to anti-inflammatory M2 phenotype and enhance the intestinal mucosal barrier in DSS-induced UC mice. Additionally, RAW264.7 cells stimulated with LPS were employed to confirm the reversal of miR-31-5p antagomir’s therapeutic effect under AMPK inhibition. The findings demonstrated that miR-31-5p antagomir penetrated colonic tissues and ameliorated DSS-induced experimental colitis. Transformation of spleen and mesenteric lymph node macrophages from M1 to M2 type was seen in the DSS+miR-31-5p antagomir group. AMPK/Sirt1 expression increased while NLRP3 expression decreased. Expression of M2-related genes and proteins was enhanced and that of the M1 phenotype suppressed. Tight junction proteins, ZO-1 and occludin, were increased. The therapeutic effects of miR-31-5p antagomir transfection into RAW264.7 cells were repressed when AMPK expression was inhibited. Therefore, our results suggest that suppression of miR-31-5p expression transformed macrophages from M1 to M2, ameliorated inflammation and repaired the intestinal epithelium to alleviate DSS-induced colitis. AMPK/Sirt1/NLRP3 was involved.

## INTRODUCTION

Ulcerative colitis (UC) is characterized by long-term inflammation and ulcers of the rectum and colon, severe diarrhea, abdominal pain, blood in the stool and weight loss. It is considered to be caused by complex interactions among environmental factors, genetic susceptibility, changes to the gut microbiota and immune imbalance of the intestinal mucosa. There is no cure and the condition is ameliorated by treatment with cortisol-based drugs, salicylic acid preparations, immunosuppressive agents and traditional Chinese medicine. Surgery is rarely employed in the absence of specific indications [1]. Immune imbalance is central to UC pathogenesis. Macrophages respond to pathogen invasion and the tissue microenvironment, helping to re-establish homeostasis [2]. UC-related inflammation stimulates reactive oxygen species (ROS)-production in the intestinal lumen which is decreased by AMPK-inhibition of the NFκB signaling pathway, preventing oxidative stress damage. AMPK is also a cellular energy sensor and ensures an energy supply for macrophage activation [3]. Indeed, a high potency of AMPK is associated with a reduced pro-inflammatory macrophage state and involvement of the downstream signaling molecules in macrophage polarization may be relevant to UC [4].

MicroRNAs (miRNAs) are small noncoding RNA molecules of 20-24 nucleotides in length which complex with RNA-induced silencing complex (RISC) to block translation of target genes and their downstream biological processes [5]. The identification of miRNAs targeted to the AMPK gene would allow manipulation of AMPK expression and regulation of the macrophage inflammatory state in UC. MiRNAs differentially expressed in colon samples from UC patients and healthy controls were identified from a miRNA microarray that Integrated human and mouse UC datasets. MiRNAs directed against AMPK were found in the TargetScan, miRDB and miRTarBase databases [6]. Intersection of the differentially expressed and anti-AMPK miRNAs was used to construct a nomogram. Finally, miR-31-5p, which inhibits the Cab39/AMPKα pathway and causes aggravated LPS-induced lung injury, selected for further study [7]. Toyonaga et al. has indicated the upregulation of miR-31-5p in inflammatory bowel disease and its association with colonic epithelial cell integrity and function [8]. It is hypothesized that administration of an miR-31-5p inhibitor or antagomir may up-regulate AMPK expression, restore macrophage polarization and repair intestinal epithelial damage, improving UC.

## MATERIALS AND METHODS

### Microarray analysis

The miRNA expression profile GSE155302 in UC mice was obtained from the Gene Expression Ominibus (GEO) database, which was composed of colon and blood of 3 dextran sulfate sodium mouse models of acute ulcerative colitis and 3 healthy controls. By integrating human and mouse UC datasets, GSE155302 presented a panel of 51 differentially regulated genes in UC colon and blood that can improve molecular phenotypes and aid in therapeutic decision-making, drug discovery and clinical trial design. Here, standardized colon group and healthy group data were selected for analysis.

### Differential expression miRNAs targeting AMPK in UC were screened

After GSE155302 standardized data were downloaded, the differentially expressed miRNAs (DEmiRNAs) between UC colon and control colon were screened out using Bioconductor limma R, with the thresholds of | log2 (fold-change) | > 1.0 and adjusted *P*-value < 0.05. The differential miRNAs were then mapped using pheatMap and GGPlot packages. Cytoscape (version 3.9.1) was used to visualize miRNA-AMPK network. A Perl program based on miRDB, miRTarBase and TargetScan databases was used to identify miRNAs targeted to the AMPK gene in question [9]. DEmiRNAs were intersected with miRNAs that targeted AMPK to obtain differential miRNAs that could target AMPK in the UC colon.

### Clinical prediction models were established for overlapping miRNAs

The least absolute shrinkage and selection operator (LASSO) was used to decrease overlapping miRNAs. LASSO used GLMNET/R packages to screen out the genes with the best diagnostic features and selected the features with non-zero coefficients in the LASSO regression model. A nomogram would be used to establish a model for diagnosing UC using miRNA. Each miRNA would intersect the upper scale with a vertical line upwards, and the scores would be added up to discover the total score on the lower scale. The vertical line would intersect the probability of diagnosing UC with miRNA. In order to determine the efficacy of miRNA in diagnosing UC, the area under receiver operating characteristic (ROC) curve and C-index were used to verify the prediction impact of the model, and decision curve analysis (DCA) was used to calculate the benefit of the prediction model. DCA is a decision curve analysis to confirm the clinical usefulness of the rosette by quantifying the net benefits under different threshold probabilities. This analysis determined the net benefit of the prevalence of UC diagnosed with miRNA at each threshold probability, and *P* < 0.05 was considered statistically significant.

### Animals and drugs

Male SPF C57BL6/J mice, 6–8 weeks old, were provided by Experimental Animal Center of Huazhong University of Science and Technology (HUST, China) (Quality Certification of Laboratory Animals: SCXK(j)2021-0006) (Ethics number: S2889), which were kept in the experimental animal center of HUST. They are housed at room temperature (22–24°C) and have free access to food and water. In this research, all animal experiments were conducted in accordance with the guidelines of the HUST Institute of Animal Research Committee and approved by the HUST Institutional Animal Care and Use Committee. Mice miR-31-5p antagomir, the sequence of which was 5′-CAGCUAUGCCAGCAUCUUGCCU-3′, and negative control (NC) antagomir were purchased from Tsingke Biotechnology Co., Ltd. (Beijing, China). Both are labeled with cholesterol modification at the 3′ end, two exoskeleton modifications at the 5′ end, four exoskeleton modifications at the 3′ end, and full-chain methoxyl modification. The miR-31-5p antagomir of 2OD and NC antagomir of 2OD showed fluorescence modification at the 5′ end. The miR-31-5p antagomir was designed by Tsingke Biotechnology Co., Ltd. (Beijing, China). This product has been widely used in basic research and has been featured in multiple publications. It is an accurately sized and chemically modified single-stranded miRNA antagomir. The miRNA antagomir undergoes purification and analysis by HPLC, resulting in a purity of >95%. It is non-toxic and meets safety standards.

### Mouse models of colitis and treatments

All mice acclimated to the environment for 7 days. The 40 mice were then randomly divided into five groups, each containing 8 mice: Normal control group (normal group), Dextran sodium sulfate (DSS) induced model group (DSS group), miR-31-5p antagomir group, negative control group (NC antagomir group), and positive control group (dexamethasone injection group). Mice were given 3.0% DSS (36–50K DA; MP Biomedicals, Irvine, CA, USA) fresh drinking water for 7 days, except for the normal group. In the meantime, starting on the fifth day, the miR-31-5p antagomir and NC antagomir groups were treated by intraperitoneal injection of miR-31-5p antagomir (Tsingke) and corresponding negative control (Tsingke) using a 1ml syringe at 3.3 mg/kg (2OD) daily dose for three consecutive days. The antagomir is cholesterol-modified at the 3′ end, bearing two exocyclic modifications at the 5′ end, and four exocyclic modifications at the 3′ end, along with full-chain methoxy modification. These modifications serve to enhance the *in vivo* stability of antagomir, enabling it to exert a sustained biological effect without inducing undesirable side effects. The *in vivo* experimental administration method of antagomir is as previously described [10]. The other groups were given equal volumes of PBS. The dexamethasone group was treated with 1 ml syringe intraperitoneal injection of 1.5 mg/kg dexamethasone injection daily.

### Evaluation of the severity of modeling in mice

Clinical manifestations of mice (*n* = 8/group) were monitored daily during modeling. In simple terms, the animal’s weight loss, fecal consistency, and presence of blood in the stool were assessed and recorded daily for a Disease Activity Index (DAI) score. All groups were sacrificed by cervical dislocation on day 8. Their colons were dissected lengthwise from the ileocecal area to the distal rectum and gently rinsed in cold phosphate buffered saline to remove feces. The colons were removed and measured for length. Histological analysis of colon tissue from each mouse was then performed. These specimens were fixed in formalin buffer, paraffin-embedded, 5-micron sections, and stained with hematoxylin and eosin (HE). Intestinal inflammation with HE staining was graded in a blind manner. In short, four parameters (depth of ulcer, extent of ulcer, presence of inflammation, and location of fibrosis) were used to calculate the histological score. Each individual parameter was rated from 0 to 4 according to the seriousness of colon changes: no change was rated 0; the minimum change score was 1; the mild shift score was 2; the moderate change score was 3; severe change was rated 4. The lowest score was 0, implying no inflammation. The higher the total score of the four parameters, the more severe the inflammation of the colon tissue [11].

### Immunofluorescence of frozen sections

After the mice were sacrificed, colon tissue was dissected longitudinally to remove intestinal feces, and then frozen in liquid nitrogen for 5 min. The tissue was embedded by OCT and sectioned with a thickness of 8-10 um. The slices were labeled. Frozen sections were rewarmed at room temperature for 30 min and fixed with 4% paraformaldehyde for 20 min. They were then placed in a repair box filled with EDTA antigen repair buffer (PH 8.0) in a microwave oven for antigen repair. After the circle was shut, the primary antibody was added and incubated at 4°C overnight. The second antibody was added the next day to re-stain the nucleus with DAPI. Finally, the film was sealed for microscopic examination and photography. The DAPI stained nucleus was blue under UV excitation, and the positive expression was corresponding fluorescein labeled red.

### *In situ* hybridization

The cell slides and frozen sections were fixed in 4% paraformaldehyde containing 1/1000 depC for 10-15 min, then rinsed with PBS. After the tissue cells were restored to water, the drilling solution was dropped to enable the miR-31-5p probe to penetrate the cell membrane quickly and smoothly. The sections after *in situ* hybridization were placed under fluorescence microscope to observe the red fluorescence intensity in colon tissue. Appropriate regions were selected and appropriate magnification was used to take photos. The fluorescence images were taken and the red fluorescence intensity in the *in situ* hybridization section of mouse colon tissues was counted by ImageJ software, so as to detect the expression level of miR-31-5P in the colon tissues of mice in each group.

### Enzyme-linked immunosorbent assay (ELISA)

Blood was collected from eyeballs of mice. After centrifugation, the supernatant was collected to detect the expression of IL-1β, TNF-α, TGF-β and IL-10. The sandwich enzyme immunoassay technique was used and the 450 nm OD was measured using a microplate reader based on the manufacturer’s protocol. According to the absorbance value of the standard substance of the gradient diluted cytokine, the standard curve was drawn and the concentration of the sample was calculated.

### Immunofluorescence (IF) and immunohistochemical staining (IHC)

Immunohistochemistry [12] and immunofluorescence staining [13] were performed as described in the literature with minor adjustments. In brief, paraffin embedded sections of 5-micron thick colon tissue samples were dewaxed and dehydrated in ethanol solutions of different concentrations, then antigen repaired, circled autofluorescence quenched, and serum blocked. The primary immunofluorescence antibodies were incubated with ZO-1 (1:300, 21773-1-AP, Proteintech, China) and occludin (1:2000, 66378-1-Ig, Proteintech), and the secondary antibodies were incubated with FITC-labeled goat anti-mouse antibody (1: 200, B100803, Baiqiandu, China), and FITC-labeled goat anti-rabbit antibody (1:200, B100805, Baiqiandu).

Primary antibodies for immunohistochemistry were incubated with P-AMPK (1:100, AF3423, Affinity Biosciences, USA), SIRT1 (1:100, A17307, ABclonal, USA), NLRP3 (1:100, A12694, ABclonal), and the secondary antibody was incubated with horseradish peroxidase (HRP) labeled sheep anti-rabbit antibody (1:2000, 5220-0336, SeraCare, USA). The nuclei were counterstained, dehydrated and sealed, and the images were observed and collected under a positive fluorescence microscope (Nikon Eclipse C1, Japan). The positive expression in immunofluorescence was the corresponding fluorescein labeled red or green light, and the positive expression in immunohistochemistry was the corresponding brown or tan light.

### QRT-PCR for miRNA and mRNA

The mRNA levels of miR-31-5p and AMPK, Sirt1, NLRP3, caspase-1, NOS2 and Arg1 were measured by qRT-PCR. U6 and GAPDH were used as reference controls. The sequences of primers are listed in Table 1.

**Table 1:** Primer sequences used for quantitative real-time PCR.

**Gene name**		**Primer sequences (5′ to 3′)**
U6	Forward	CGCTTCGGCAGCACATATAC
	Reverse	CACGAATTTGCGTGTCATCC
GAPDH	Forward	ACTCTTCCACCTTCGATGCC
	Reverse	TGGGATAGGGCCTCTCTTGC
miR-31-5p	Forward	AATTAAGGCAAGATGCTGGCA
	Reverse transcription	GTCGTATCGACTGCAGGGTCCGAGGTATTCGCAGTCGATACGACCAGCTA
	common-R	ACTGCAGGGTCCGAGGTATT
AMPK	Forward	CACTGACTTTATCCTGGTGCTGC
	Reverse	GGAACTTGAGTAGCCGCTTGTG
Sirt1	Forward	TGCTGGCCTAATAGACTTGCAAA
	Reverse	ATACCTCAGCACCGTGGAATATG
NLRP3	Forward	TATCTCTCCCGCATCTCCATTTG
	Reverse	GCGTTCCTGTCCTTGATAGAGTA
Caspase-1	Forward	TTCAACATCTTTCTCCGAGGGTT
	Reverse	CACCTCTTTCACCATCTCCAGAG
NOS2	Forward	TTTGCTCATGACATCGACCAGAA
	Reverse	CGTTTCGGGATCTGAATGTGATG
Arg1	Forward	TCTGCCAAAGACATCGTGTACAT
	Reverse	CGACATCAAAGCTCAGGTGAATC

### Western blot

As mentioned above [14], Western blot analysis was used to quantify protein expression levels in colon tissues. The primary antibodies and their dilution concentrations were as follows: Phospho-AMPK (1:1000, AF3423, Affinity Bioscience), AMPK (1:5000, 10929-2-AP, Proteintech), SIRT1 (1:1000, A17307, ABclonal), NLRP3 (1:2000, A12694, ABclonal), caspase-1 (1:1000, A16792, ABclonal), iNOS (1:1000, A3200, ABclonal), Arg1 (1:1000, A1847, ABclonal), NOX2 (1:1000, A19701, ABclonal), and HADHB (1:1000, A5716, ABclonal). β-actin (1:1000, GB12001, Servicebio, China) was used as the internal reference. HRP Goat Anti-Rabbit IgG (1:10 000, AS014, ABclonal) was used as the secondary antibody. We used ImageJ Software to analyze the protein band results.

### Flow cytometry

The spleens and lymph nodes of mice were isolated, and the cells were passed through a 70-um cell filter by kneading the tissue with a syringe plunger to make a single cell suspension. We first incubated cells with KO525/BV510-anti-FVD (423101, Biolegend, USA), APC-Cy7-anti-CD45 (103115, Biolegend), PC-Cy5.5-anti-CD11b (101227, Biolegend), PE-anti-F4/80 (157303, Biolegend) and BV421-anti-CD11c (117329, Biolegend) antibodies for 30 min at 4°C in the dark. For intracellular staining, cells were further fixed, permeated with Fix/Perm buffer, and then stained with APC-anti-CD206 (141707, Biolegend) antibody for 40-50 min at room temperature. Finally, the stained cells were analyzed by flow cytometry.

### Dual luciferase reporter assay

AMPK wild-type 3′uTRs and mutant 3′UTRs were cloned into pmirGLO vector, and MMU-miR-31-5p or control miRNA were mixed with Lipo2000 and co-transfected into 293T cells in good condition, respectively [15]. Luciferase activity was measured by Dual-Luciferase^®^ Reporter Assay System (E1910, Promega, USA) kit.

### Cell culture and inflammatory induction

RAW264.7 cell line was purchased from American Type Culture Collection (ATCC, USA) and cultured in DMEM (Gibco, USA) supplemented with 10% FBS (Gibco), 100 U/ml penicillin, and 100 μg/ml streptomycin (HyClone, USA) at 37°C in a humid 5% (v/v) CO2 atmosphere. The culture medium should be changed regularly according to the growth of the cells, and when the cells grew to 70–80% confluence, they can be used for relevant experimental studies. Cell models were induced with 1000 ng/ml LPS for 12 h as previously described [16], and cells were treated with 10 μM AMPK inhibitor (Dorsomorphin, hy-13418, MCE, USA) for 12 h prior to model induction according to pre-experiments.

### Cell transfection

According to the manufacturer’s protocol, 12 h after Dorsomorphin intervention, miR-31-5p antagomir (2 nM) and NC antagomir (2 nM) were transiently transferred into RAW264.7 cells utilizing TSnanofect (1 mg/ml) reagent. After 48 h of transfection, the inflammatory model was induced by LPS. Cells were harvested 12 hours after modeling for subsequent tests.

### Statistical analysis

Data are expressed as mean standard deviation (SD). Multiple comparisons were performed by one-way ANOVA using GraphPad Prism 8 followed by Tukey’s posttest. *P*-values < 0.05 were considered statistically significant.

### Data availability

Data will be made available on request.

## RESULTS

### miR-31-5p is a clinically significant non-coding RNA that targets AMPK and is highly expressed in humans and mice with ulcerative colitis

**Figure 1:**
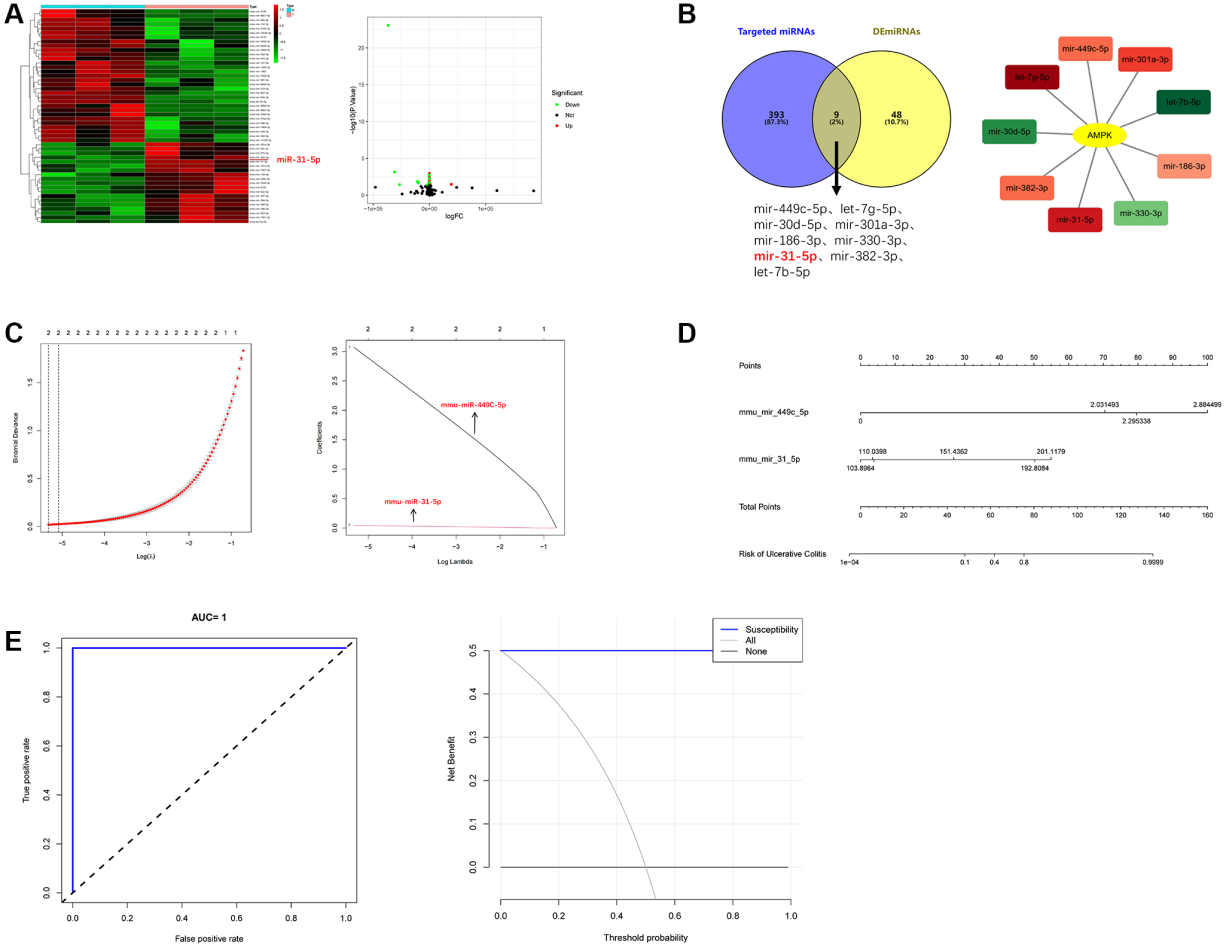
**The highly expressed miRNA molecules that can target AMPK in UC were systematically screened.** (**A**) Heat map and volcano map of DEmiRNAs. (**B**) Intersection diagram and cytoscape diagram of DEmiRNAs and AMPK-targeting miRNAs. (**C**) Path compression diagram and model diagram of LASSO regression. (**D**) Diagnostic model of mmu-miR-449C-5p and mmu-miR-31-5p (nomogram). (**E**) ROC curve and DCA curve of model validation.

Differentially expressed miRNAs were obtained from the sequencing data of colon samples in GSE155302 data set in GEO by bioinformatics analysis, as shown in Figure 1A. These DEmiRNAs were further intersected with miRNAs that could target AMPK found in miRDB, miRTarBase and TargetScan databases. Cytoscape showed the targeting relationship between 9 intersection miRNAs and AMPK (Figure 1B).

Of the 9 core miRNAs molecules, mmu-miR-449C-5p and mmu-miR-31-5p were selected as the best predictive molecules by LASSO regression (Figure 1C). Nomogram showed that the higher the scores of mmu-miR-449C-5p and mmu-miR-31-5p, the higher the risk of being diagnosed with UC (Figure 1D). The C-index showed that the model discrimination performance was 1, the area under the receiver operating characteristic curve (ROC) was equal to 1, and the clinical decision curve (DCA) showed two molecules of nomogram to diagnose UC had an additional benefit over all UC or all non-UC diagnoses [17]. Therefore, the internal verification of the model proved the correctness of the model (Figure 1E). Since the difference factor of miR-31-5p was more significant, this study took miR-31-5p as the research focus.

### miR-31-5p antagomir decreased the level of miR-31-5p in DSS-induced colitis

**Figure 2:**
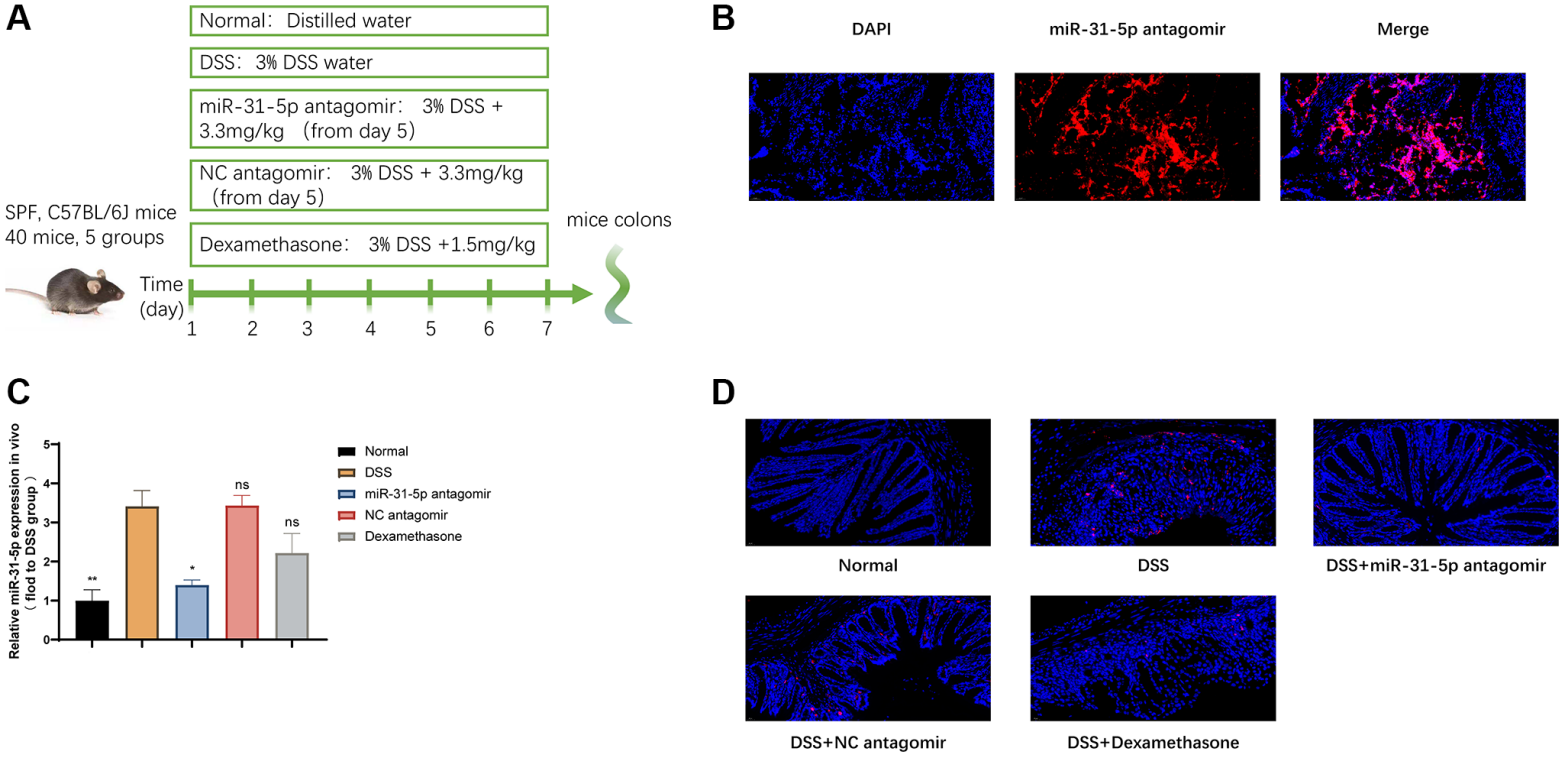
**miR-31-5p antagomir decreased the level of mir-31-5p in DSS-induced colitis.** (**A**) Diagram of drug intervention process in animal experiments. (**B**) Immunofluorescence of frozen sections showed the localization of miR-31-5p antagomir in mouse colon. (**C**) QRT-PCR was used to analyze the level of miR-31-5p in colon tissues of mice. Each column represents the mean ± SD, *n* ≥ 3 from each group. ns represented *P* > 0.05, ^*^P < 0.05, ^**^P < 0.01 vs. DSS group. (**D**) *In situ* hybridization analysis of miR-31-5p in mouse colon. Red represented positive signal for miR-31-5p (magnification ×400).

We tested the effect of miR-31-5p antagomir reaching the colon of mice by intraperitoneal injection. Figure 2B showed that miR-31-5p antagomir can locate in colonic cells. Subsequent animal experiments were carried out, and the procedure was shown in Figure 2A. We quickly used qPCR assay to find that the expression of miR-31-5p in colon tissues induced by DSS was increased compared with the normal group, and the level of miR-31-5p was significantly decreased by antagomir compared with the DSS group (*P* < 0.05) (Figure 2C). Moreover, fluorescence *in situ* hybridization experiment confirmed that miR-31-5p was typically distributed in colon tissue cells, and the positive expression of miR-31-5p was increased in the DSS group compared with the normal group, while the use of antagomir of miR-31-5p significantly reduced the elevation of miR-31-5p caused by DSS (*P* < 0.01) (Figure 2D). The above results indicated that the level of miR-31-5p in DSS-induced colitis colonic tissue was increased, which was consistent with the results of bioinformatics analysis, and miR-31-5p antagomir can reduce miR-31-5p levels in colonic tissue of mice.

### High expression of miR-31-5p is one of the key factors in DSS-induced colon damage in mice

**Figure 3:**
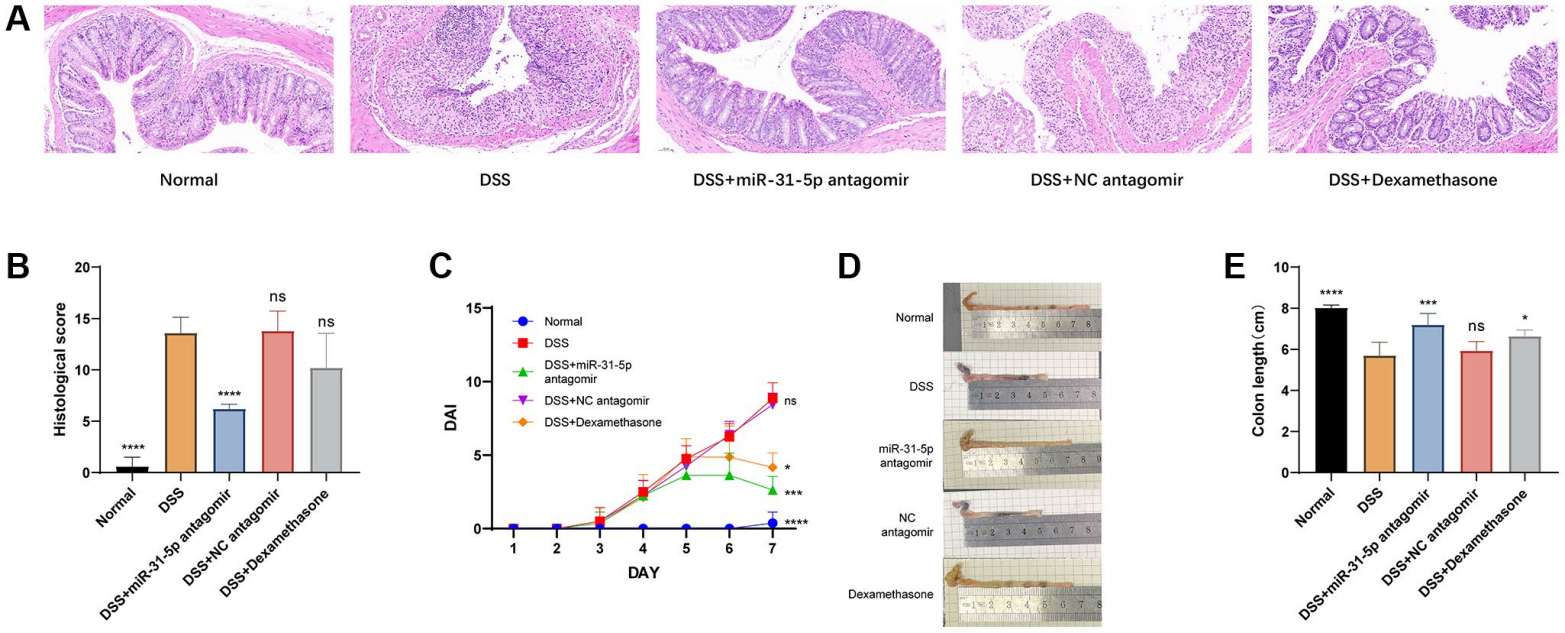
**miR-31-5p antagomir contributed to the damage of DSS to colon in mice.** (**A**) Analysis of hematoxylin and eosin (HE) from colonic samples (x200 magnification). (**B**) DAI scores. (**C**) Colon lengths were presented. (**D**) Statistics of colon length. (**E**) Histological scores based on HE staining method. Each bar represents mean ± SD, *n* ≥ 3 from each group. ns represented *P* > 0.05, ^*^P < 0.05, ^**^P < 0.01, ^***^P < 0.001, ^****^P < 0.0001 vs. DSS group.

In this study, the body weight, fecal viscosity, hematochezia, and general condition of mice were recorded daily. DSS-induced mice presented significant loss of appetite and weight, severe diarrhea and severe mucous, pus, and blood stool, reduced activity, and generally poor condition. The histopathological score of HE was performed in a blind way [18]. Compared with the DSS group, it was found that the use of miR-31-5p antagomir reduced the score by 54% (*P* < 0.0001), while the use of Dexamethasone only reduced the score by 25% (Figure 3B). Dexamethasone is commonly used for the treatment of colitis in mice and humans. In this study, it was employed as a positive control reagent for mice and administered via intraperitoneal injection [19]. We found that compared with the DSS group, the administration of miR-31-5p antagomir significantly reduced the DAI score [20] (*P* < 0.05) (Figure 3C). With symptoms of bloody diarrhea and weight loss, colon length was significantly shorter in the DSS group than in the normal group, which was alleviated by miR-31-5p antagomir (*P* < 0.001) and Dexamethasone (Figure 3D, 3E). In the most intuitive colonic pathological HE staining, we observed considerable area necrosis, mucosal edema, goblet cell depletion, the disappearance of glandular structure, and inflammatory infiltration in the colon tissue of mice in the DSS group. The negative control of miR-31-5p antagomir was almost consistent with that in the DSS group, which was in sharp contrast to the normal group (Figure 3A). Therefore, our results proved that miR-31-5p was involved in colonic inflammation, and miR-31-5p antagomir could reach colonic tissues to alleviate DSS-induced colonic immune inflammation in mice.

### miR-31-5p antagomir can reduce intestinal mucosal inflammation and repair intestinal mucosal barrier function in DSS mice by down-regulating miR-31-5p

**Figure 4:**
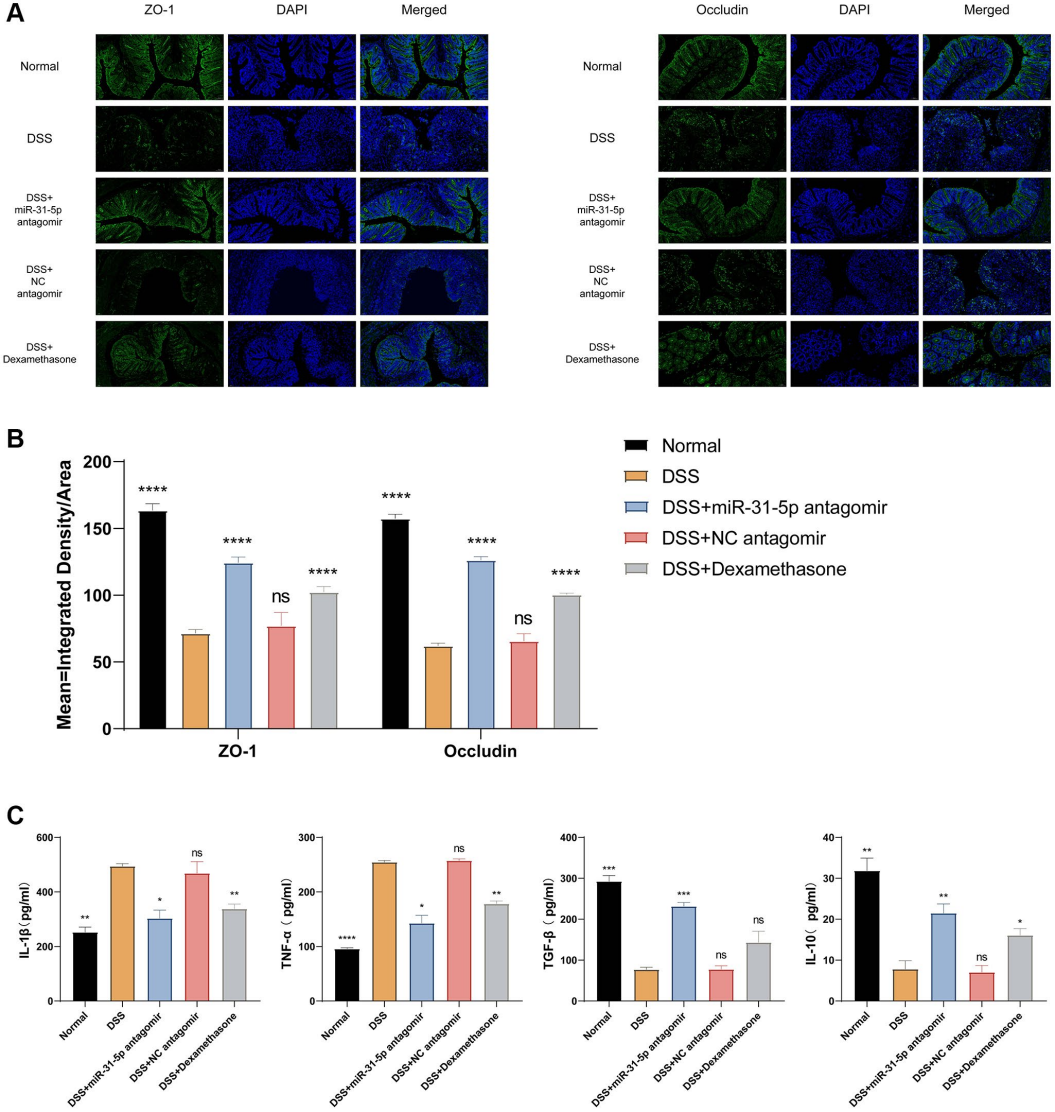
**mir-31-5p antagomir restored intestinal mucosal tight junction protein and reduced colonic inflammation in DSS-induced colitis mice.** (**A**) Immunofluorescence staining of ZO-1 and occludin in colon tissue (x200 magnification). (**B**) Quantitative fluorescence analysis of ZO-1 and occludin (ordinate represented the average fluorescence intensity analyzed by ImageJ software). (**C**) ELISA analysis of the expression of IL-1β, TNF-α, TGF-β and IL-10 in colon. Each bar represents mean ± SD, *n* ≥ 3 from each group. ns represented *P* > 0.05, ^*^P < 0.05, ^**^P < 0.01, ^***^P < 0.001, ^****^P < 0.0001 vs. DSS group.

As previously described, miR-31-5p antagomir can be intraperitoneally injected into colonic tissues of mice, and miR-31-5p is highly expressed in colitis. To understand the potential therapeutic role of miR-31-5p antagomir, we performed immunofluorescence assay on colonic tight junction proteins. As shown in Figure 4A, the levels of zonula occludens-1 (ZO-1) and occludin proteins were decreased in the DSS group compared with the normal group (*P* < 0.0001). Antagomir treatment can reduce Mir-31-5p and restore the expression of these tight junction proteins (*P* < 0.0001). Quantitative fluorescence analysis of ZO-1 and occludin showed that the treatment of miR-31-5p antagomir, to some extent, could significantly improve the tight junction protein level and alleviate the damage of epithelial barrier function in colon tissue caused by DSS (Figure 4B).

Apart from detecting tight junctions, we also measured inflammatory and anti-inflammatory cytokine levels in the serum of mice by ELISA assay as a proxy for the intestinal inflammatory status of mice in different treatment groups. We can see that the proinflammatory cytokines IL-1β and TNF-α were increased in the DSS group, while the effect of antagomir of miR-31-5p can reduce their expression. On the contrary, the anti-inflammatory cytokine IL-10 decreased in the DSS group, the intervention of miR-31-5p antagomir could restore its expression, and the expression of TGF-β showed a similar trend to that of IL-10 (Figure 4C).

### The expression of miR-31-5p is negatively correlated with the expression of AMPK in DSS-induced colonic tissues in mice

**Figure 5:**
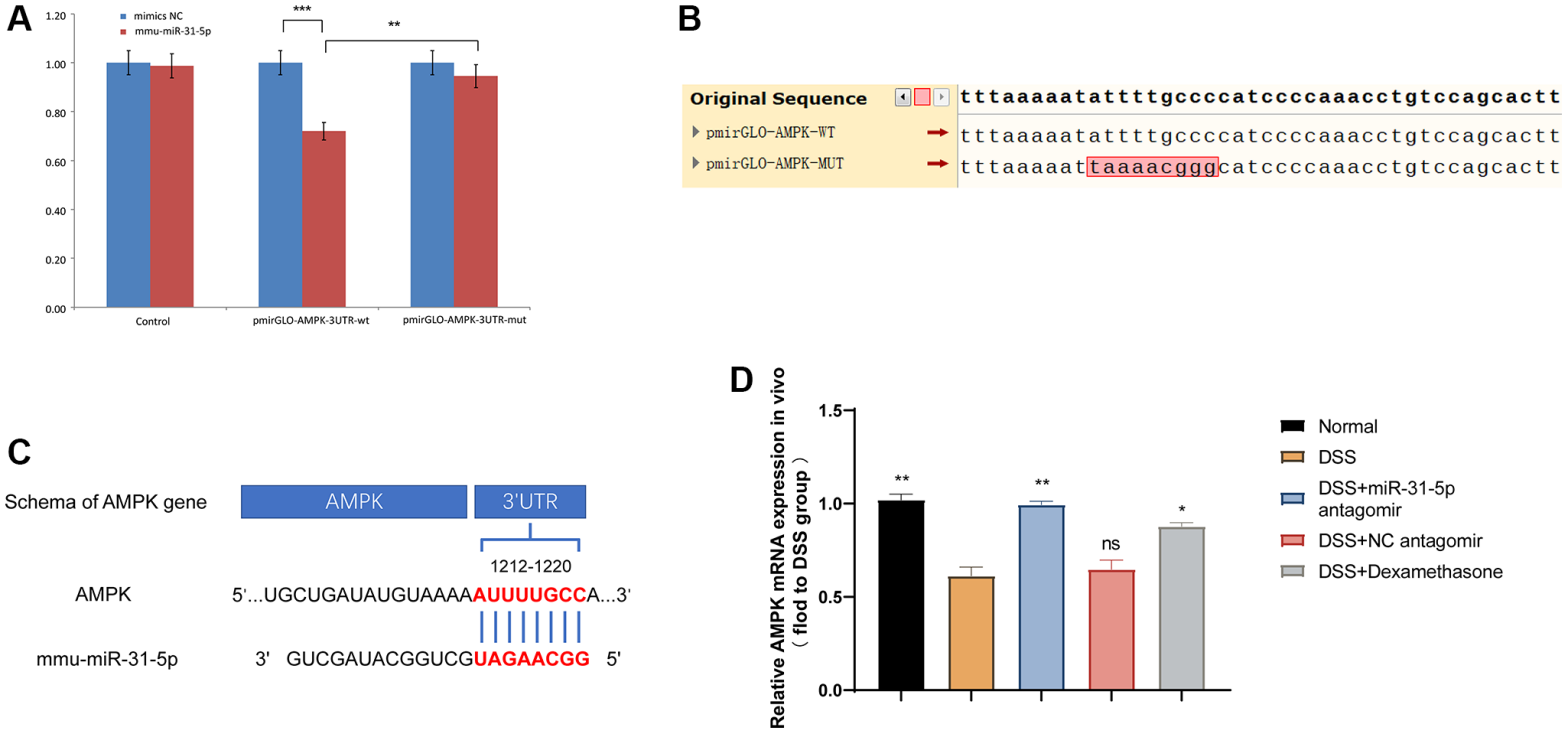
**miR-31-5p can target AMPK in mouse colon tissues.** (**A**) Dual luciferase report assay of AMPK 3′UTR wild-type or mut-type and miR-31-5p. ^**^P < 0.01, ^***^P < 0.001 vs. Negative Control. (**B**) Sequence difference between two cloned plasmids: the difference is located in the cloned AMPK DNA fragment, in which the sequence ATTTTGCCC in pmirGLO-AMPK-WT corresponding to the red box is the targeting fragment of mir-31-5p. (**C**) Wild-type sequences of AMPK for miR-31-5p target. (**D**) Relative mRNA of AMPK in mice colonic tissue was measured by qPCR (*n* ≥ 5). Each bar represents mean ± SD. ns represented *P* > 0.05, ^*^P < 0.05, ^**^P < 0.01, ^***^P < 0.001 vs. DSS group.

As mentioned above, miR-31-5p is involved in the destruction of colon tissue by DSS in mice, and the use of antagomir of miR-31-5p seemed to alleviate this process. Therefore, we used dual luciferase reporter assay and qPCR to explore the targeting relationship between miR-31-5p and its downstream AMPK. After mmu-miR-31-5p intervention, AMPK-3′UTR activity decreased by 28%, indicating that mmu-miR-31-5p acted on the AMPK-3′UTR region (*P* < 0.01), while AMPK-3′UTR mutated by mmu-miR-31-5p increased the activity of the mutant 3′UTR by 23% compared with the wild-type (*P* < 0.01) (Figure 5A). Figure 5B showed the site of the gene mutation. The results confirmed the direct interaction between miR-31-5p and AMPK (Figure 5C). Subsequently, we quantified AMPK mRNA levels in colitis tissues using qPCR and found that AMPK mRNA levels in DSS group were decreased compared with normal group and increased by miR-31-5p antagomir (Figure 5D). It was further confirmed that miR-31-5p could target AMPK in colon tissues, and miR-31-5p antagomir could reverse this process.

### miR-31-5p can target the AMPK/Sirt1/NLRP3 axis in DSS-induced mice colitis

**Figure 6:**
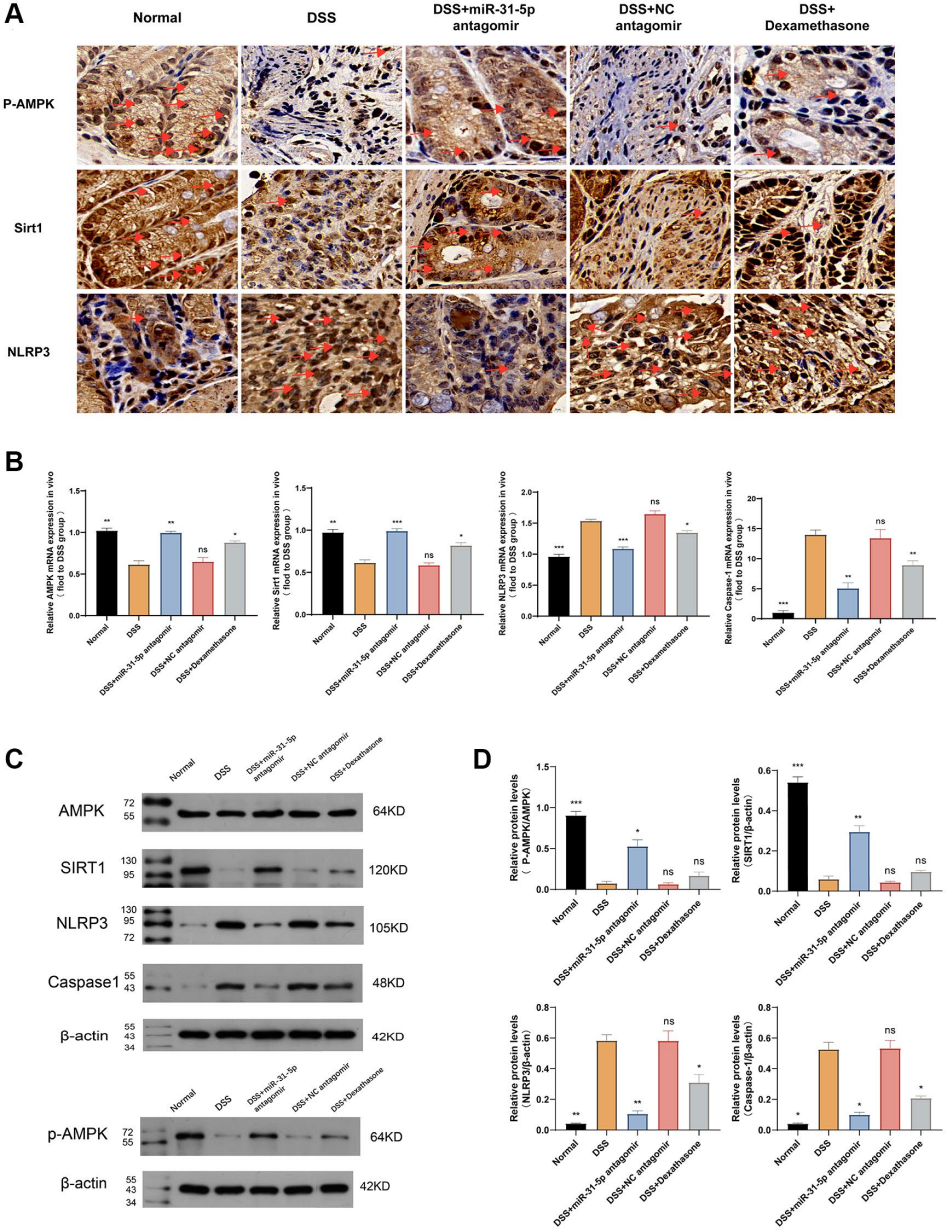
**mir-31-5p may target AMPK/Sirt1/NLRP3 axis to mediate DSS-induced colonic mucosal inflammation in mice.** (**A**) Immunohistochemical analysis of P-AMPK, Sirt1 and NLRP3 in colon of mice. Yellow brown were positive signals (the ruler was 10 um, and the positive signals were marked by red arrows). (**B**) The relative mRNA levels of AMPK, Sirt1, NLRP3 and caspase-1 in colon tissues of mice were detected by qPCR. (**C**) Western blot analysis of P-AMPK/AMPK, NLRP3, Sirt1 and caspase-1. (**D**) Quantitative protein analysis of P-AMPK/AMPK, Sirt1, NLRP3 and caspase-1. Each bar represents mean ± SD, *n* ≥ 5 from each group. Ns represented *P* > 0.05, ^*^P < 0.05, ^**^P < 0.01, ^***^P < 0.001 vs. DSS group.

AMPK can form a positive feedback loop with its downstream target Sirt1 to ameliorate colitis. Sirt1, a protein regulated by deacetylase, has effects in antioxidation, metabolism regulation, immunity, and enhances intestinal barrier integrity by increasing tight junction proteins [21]. Recent studies show that sirt1 reduces NLRP3 inflammasomes, improving microglia-induced inflammatory response and polarization [22]. Immunohistochemistry revealed changes in the AMPK/Sirt1/NLRP3 axis. miR-31-5p inhibited AMPK expression, leading to down-regulation of Sirt1 and increased expression of NLRP3. The use of miR-31-5p antagomir can reverse this trend (Figure 6A). In the DSS group, mRNA levels of AMPK and Sirt1 decreased compared to the normal group, but miR-31-5p antagomir intervention restored mRNA levels (*P* < 0.01) better than the dexamethasone group. NLRP3 and caspase-1, downstream inflammatory factors, increased in the DSS group, but miR-31-5p antagomir reduced their levels (*P* < 0.01) (Figure 6B). Western blotting showed decreased levels of p-AMPK (Thr172) and Sirt1 in the DSS group at the protein level, which were reversed by the addition of miR-31-5p antagomir. NLRP3 and caspase-1 showed the opposite pattern (Figure 6C). Protein quantitative analysis confirmed these findings (Figure 6D). These results suggest that the AMPK/Sirt1/NLRP3 axis may be targeted by miR-31-5p, leading to colonic mucosal inflammation in DSS-induced colitis.

### mir-31-5p antagomir specifically alleviates colitis by transforming M1-type macrophages into M2-type macrophages through AMPK pathway

**Figure 7:**
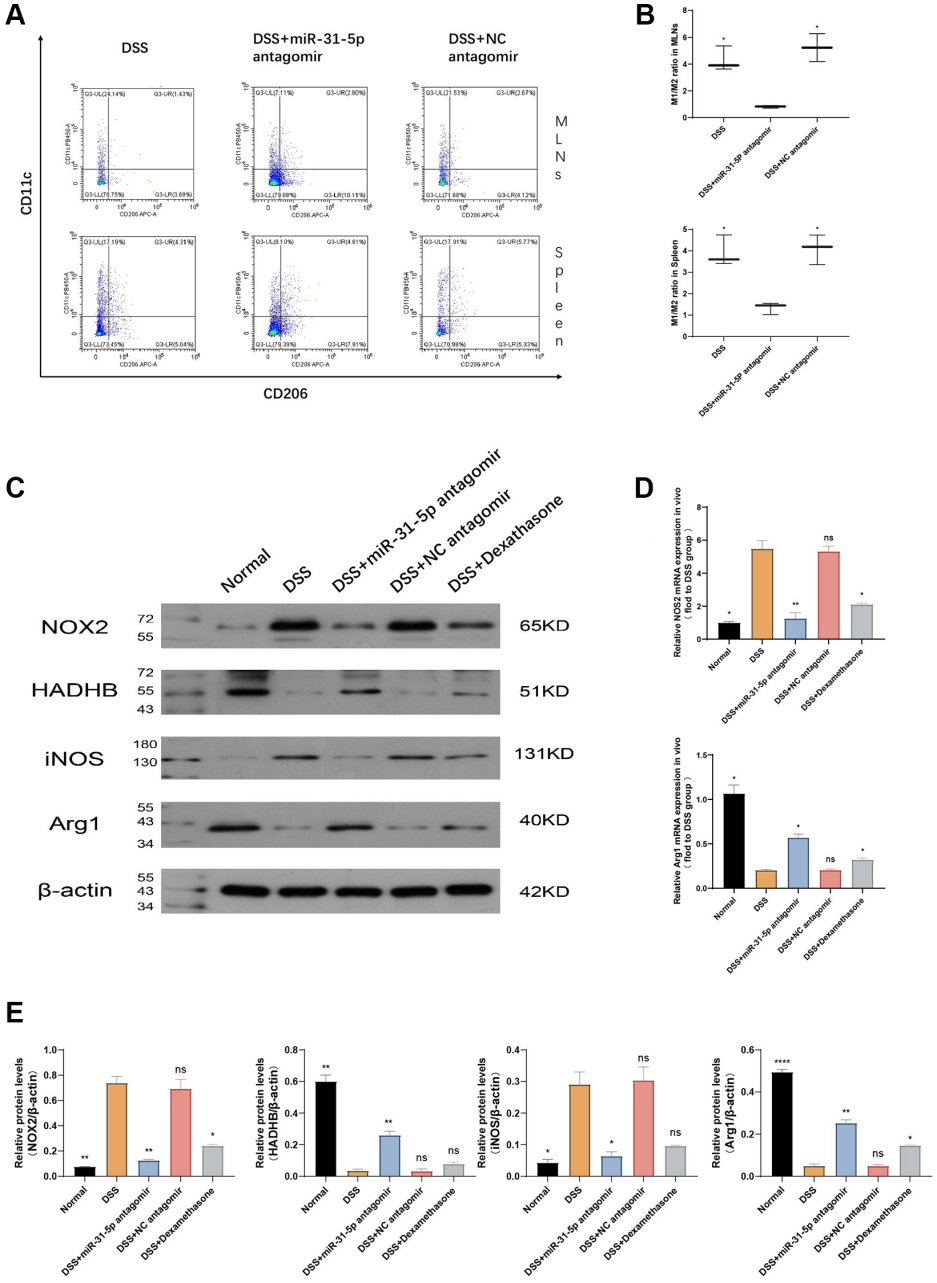
**mir-31-5p antagomir may alleviate colitis in mice by transforming M1-type macrophages into M2-type macrophages.** (**A**) The ratio of M1/M2 in spleen and lymphocytes was determined by flow cytometry. (**B**) Statistical analysis of M1/M2 ratio in spleen and lymphocytes. (**C**) The expressions of NOX2, HADHB, iNOS, Arg1 and β-catenin proteins in colon were analyzed by Western blotting. (**D**) The mRNA expression of M1-type and M2-type macrophage marker genes was detected by qPCR. (**E**) Statistical analysis of protein expression levels of NOX2/β-actin, HADHB/β-actin, iNOS2/β-actin and Arg1/β-actin. Each bar represents mean ± SD, *n* ≥ 5 from each group. Ns represented *P* > 0.05, ^*^P < 0.05, ^**^P < 0.01, ^***^P < 0.001, ^****^P < 0.0001 vs. DSS group.

Since the AMPK pathway is one of the classical pathways in macrophage polarization, we next investigated the ratio of CD45^+^F4/80^+^CD11c (M1) and CD45^+^F4/80^+^CD206^+^ (M2) in mouse spleen and MLNs nodes by flow cytometry (Figure 7A). The results showed that, compared with the DSS group, miR-31-5p antagomir could reduce the M1/M2 ratio of spleen and MLNs, which means that miR-31-5p antagomir could change the polarization state of macrophages from proinflammatory to anti-inflammatory (*P* < 0.05). The significance of flow analysis results can be seen in Figure 7B. The classic proinflammatory M1-type macrophage subtype is characterized by increased aerobic glycolysis and reduced oxidative phosphorylation (OXPHOS). In contrast, the subtype of M2-type anti-inflammatory macrophages is characterized by Fatty Acid Oxidation (FAO) and OXPHOS. HADHB is a subunit of mitochondrial trifunctional protein (MTP) that catalyzes the last three steps of fatty acid oxidation, while glycolysis increases NADPH oxidase 2 (NOX2) activity. Therefore, we analyzed the expression of HADHB protein and NOX2 by Western blotting (Figure 7C) [23]. The results confirmed the idea, compared with the normal group, NOX2 protein expression levels in the DSS group were sharply increased (*P* < 0.01), while the expression of HADHB was significantly reduced (*P* < 0.01). Our research also demonstrated that miR-31-5p antagomir can restore HADHB, a protein related to oxidative phosphorylation, and reduce NOX2, a protein related to glycolysis, to convert pro-inflammatory M1 to anti-inflammatory M2 (*P* < 0.01) (Figure 7E). It is well known that nitric oxide synthase 2 (NOS2) and Arginase 1 (Arg1) are markers of M1-type macrophages and M2-type macrophages, respectively [24]. We then verified by qPCR and WB at mRNA and protein levels, respectively, that compared with the normal group, NOS2 was increased and Arg1 was decreased in the DSS group (*P* < 0.05), and the addition of miR-31-5p antagomir could reverse this damage (*P* < 0.05), further indicating that M1 was converted to M2 (Figure 7C–7E). From the gene and protein expression analysis of M1-type and M2-type related factors, all the results emphasized that DSS caused the increase of M1 proinflammatory and the decrease of M2 anti-inflammatory in mice colon, and the intervention of mir-31-5p antagomir promoted the transformation of M1 to M2 phenotype. Our study has previously demonstrated that mir-31-5p can target the AMPK pathway and exert its damaging effect. AMPK is strongly correlated with macrophages, as well as miR-31-5p also leads to a rise in proinflammatory macrophages. Consequently, it is reasonable to assume that mir-31-5p antagomir can specifically convert M1 to M2 by the AMPK route and relieve colitis in mice.

### Inhibition of AMPK impaired the ability of miR-31-5p antagomir to transform M1-type macrophages into M2-type macrophages

**Figure 8:**
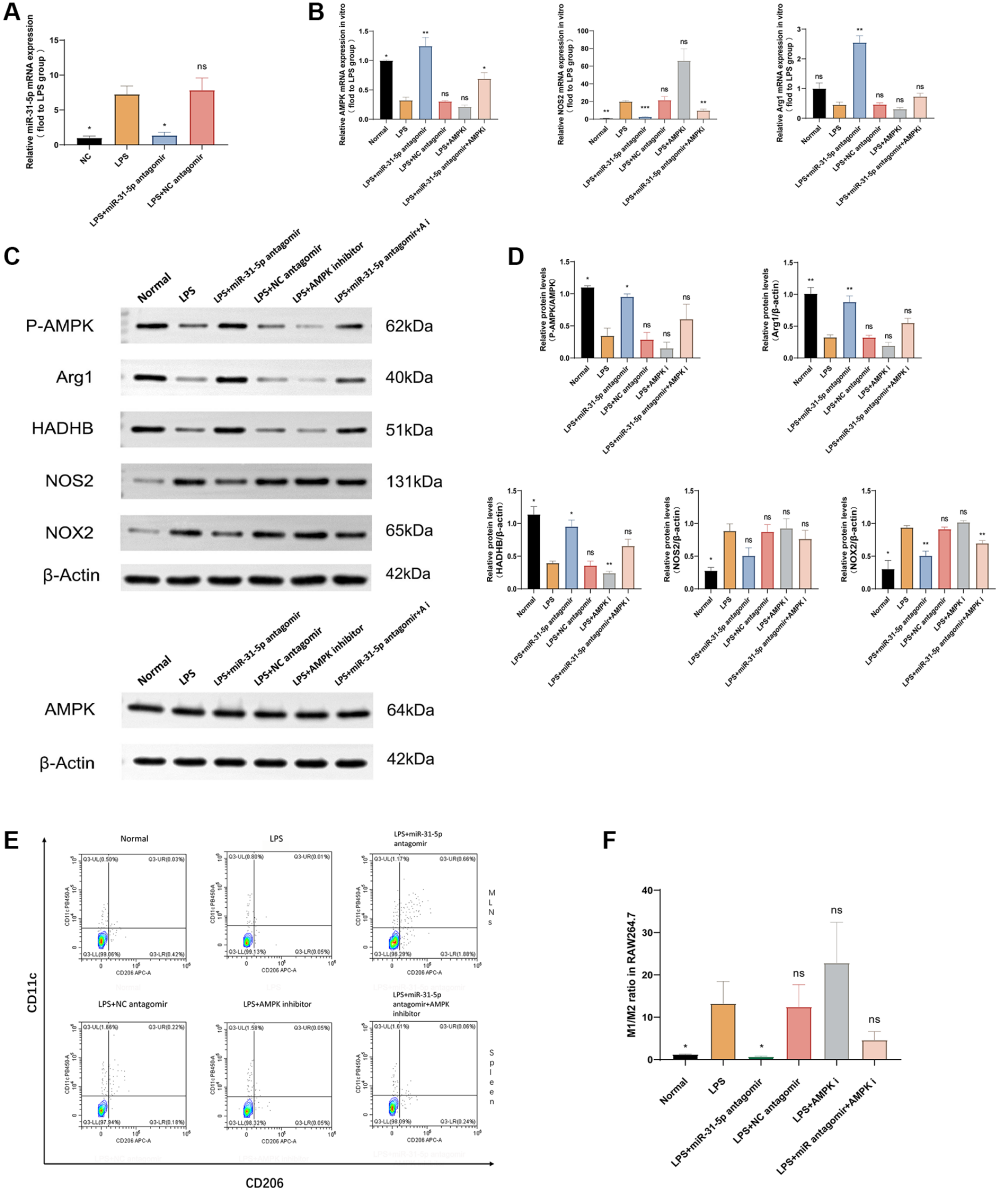
**miR-31-5p antagomir may transform macrophages from M1 type to M2 type through the AMPK-related pathway.** (**A**) miR-31-5p antagomir was successfully transfected into RAW264.7 cells. (**B**) qPCR detection of AMPK, NOS2, and Arg1 mRNAs. (**C**) Western blotting strips of P-AMPK/AMPK, NOS2, Arg1, NOX2, HADHB (β-actin). (**D**) Protein quantitative analysis of P-AMPK/AMPK, NOS2, Arg1, NOX2 and HADHB (β-actin). (**E**) Flow cytometry of M1/M2. (**F**) Statistical analysis of M1/M2 cell ratio. Each bar represents mean ± SD, *n* = 8 from each group. Ns represented *P* > 0.05, ^*^P < 0.05, ^**^P < 0.01, ^***^P < 0.001, ^****^P < 0.0001 vs. LPS group.

To explore the effect of miR-31-5p antagomir on macrophage polarization through the AMPK pathway [16], we conducted the following rescue experiment for verification. Firstly, the qPCR experiment verified that miR-31-5p antagomir could reach RAW264.7 cells through transfection, inhibit the expression of the miR-31-5p gene to play its role. From Figure 8A, we saw that compared with the normal group, the expression of miR-31-5p was significantly increased in the LPS group (*P* < 0.05), the addition of miR-31-5p antagomir can alleviate the overexpression of miR-31-5p in LPS-stimulated macrophages (*P* < 0.05). Subsequent qPCR experiments verified the expression levels of AMPK, NOS2, and Arg1 mRNAs (Figure 8B). When AMPK inhibitor was combined with miR-31-5p antagomir, compared with miR-31-5p antagomir group alone, the expression of M1-related NOS2 mRNA was increased, while the expression of M2-related Arg1 mRNA was decreased (*P* < 0.01), indicating that the therapeutic effect of miR-31-5p antagomir was limited when AMPK gene was inhibited. Then we used the WB experiment to verify that at the protein level, when the function of AMPK was inhibited, the effect of miR-31-5p antagomir on the transformation of LPS-stimulated RAW264.7 cells from M1 to M2 was also inhibited (Figure 8C). Figure 8D showed the significance of this trend by quantitative protein analysis (*P* < 0.05). Finally, we sorted M1 and M2 cell surface markers at the cellular level by flow cytometry, confirming that miR-31-5p antagomir can transform M1 inflammatory macrophages into M2 anti-inflammatory macrophages. This therapeutic effect is reduced when combined with an AMPK inhibitor, and AMPK was guaranteed to be the intermediate mediator of miR-31-5p antagomir to alter the polarization state of macrophages. (*P* < 0.05) (Figure 8E, 8F).

## DISCUSSION

Cells of both the innate and adaptive immune systems, including dendritic, B, Thelper (Th) 1, Th2, Th17 and Treg cells, are involved in UC pathogenesis. However, macrophages of the immune system are also abundant in the intestinal mucosa and the Macrophage phenotype changes (M1/M2) balance as well as related inflammatory signaling are also worthy of study with respect to UC [25]. The number of macrophages in the DSS-induced colitis mouse model vastly exceeded the number of neutrophils, indicating a greater impact of the former cell-type [26]. Macrophage plasticity allows phenotypic changes in response to environmental cues. The M1 phenotype contributes to inflammation and resists infection and the M2 phenotype has phagocytic and wound healing activity [27]. Therefore, the role of macrophages in UC is particularly important.

Interactions of intestinal macrophage and miRNA in the context of UC remain unclear. A bioinformatics approach during the current study allowed identification of miR-31-5p which is highly expressed in mouse and human colitis. MiR-31-5p expression increased in DSS-induced colonic mucosa both *in vivo* and *in vitro*, verifying the bioinformatics analysis. MiR-31-5p is overexpressed in colonic tissues of patients with IBD. Current research on miR-31-5p in the IBD gut primarily focuses on intestinal epithelial cells, where it exhibits high expression levels [28]. Our study represents the first to unveil the excessive accumulation of miR-31-5p inhibition at the macrophage level, offering insights that could contribute to alleviating colonic inflammation. The miR-31-5p antagomir reduced intestinal inflammatory injury and restored mucosal barrier function. Thus, inhibition of miR-31-5p has a potential therapeutic application for UC treatment. Antagomirs are stable and show a potent inhibitory effect, compared with other miRNA inhibitors, displacing intracellular miRNA from the targeted gene with no side effects. Use of the miR-31-5p antagomir *in vivo* significantly decreased miR-31-5p expression (*p* < 0.05) and RAW264.7 cells transfected with the antagomir *in vitro* showed suppression of miR-31-5p expression compared with LPS-treated controls (*p* < 0.05). Previous work has shown that astaxanthin downregulated miR-31-5p and M1-like proinflammatory factors to inhibit neuroinflammation [29]. Impairment of macrophage AMPK has also been associated with proinflammatory cytokine production in colitis [3, 30, 31]. Wencheng Wei et al. found that Sirt1 signaling blocked the NLRP3-initiated cascade to increase expression of tight junction proteins, occludin and ZO-1, to ameliorate colonic epithelial dysfunction in experimental colitis [32]. Furthermore, Lee et al. described the targeting of AMPK, upstream of Sirt1, by chrysanthemum extract which reduced obesity-related inflammation [33]. Loss of mitochondrial homeostasis and increased glycolysis in colitis may lead to an imbalance in the ATP/ADP ratio and activation of AMPK but this mechanism does not explain the inflammatory infiltration state of the disease [34, 35]. Sirt1 is considered a key regulator of macrophage self-renewal, integrating the cell cycle with lifespan pathways [36]. Expression of miRNA upstream of AMPK is significant given the acknowledged role of this molecule in macrophage polarization [4]. The miR-31-5p antagomir may influence macrophage differentiation and immune function through AMPK/Sirt1-dependent signaling in colitis.

**Figure 9:**
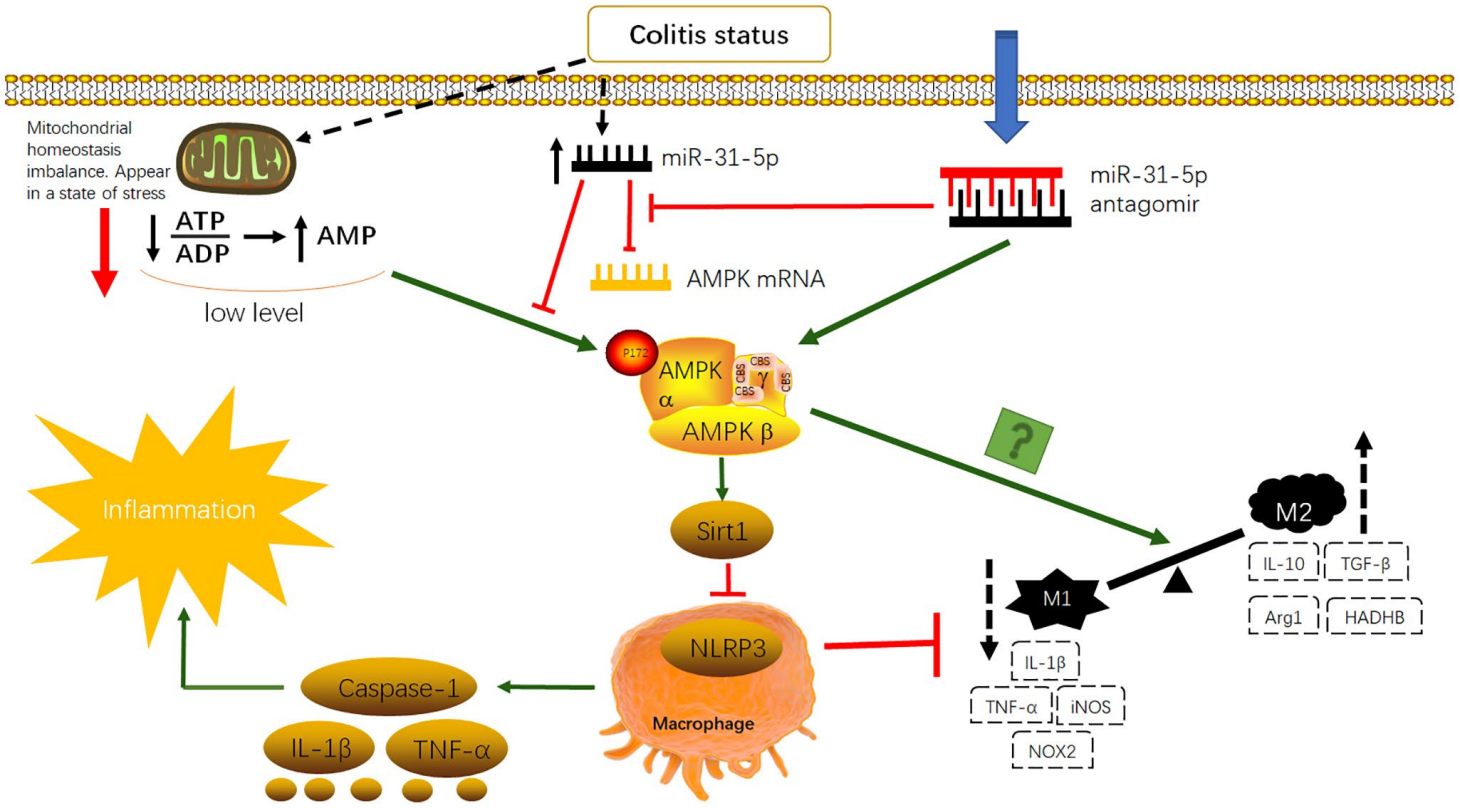
Antagomir of miR-31-5p can activate AMPK/SIRT1-dependent signaling to inhibit NLRP3 inflammasome in macrophages and promote the transformation of M1-type macrophages to M2-type macrophages to improve inflammatory response in colitis.

miR-155 has been shown to be highly expressed in UC by previous studies of our own and of others [10, 37]. However, bioinformatics analysis identified miR-31-5p to be a more likely object for study and construction of a clinical prediction model. Dual luciferase reporter assays demonstrated that miR-31-5p binds to the AMPK gene 3′-UTR (*p* < 0.05) and inhibits its expression. *In vivo* and *in vitro* experiments showed that inflammation stimulated miR-31-5p expression and that AMPK mRNA correlated negatively with miR-31-5p. AMPK may be a downstream target of miR-31-5p in UC. ZO-1 and occludin protein expression was restored after treatment with the miR-31-5p antagomir, suggesting the association of reduced miR-31-5p with the recovery of upper intestinal epithelial function [38]. Western blotting and qPCR showed decreased AMPK/Sirt1 expression after DSS treatment and use of the miR-31-5p antagomir up-regulated expression. Opposing trends were seen for NLRP3. The changes in the AMPK/Sirt1/NLRP3 pathway were accompanied by changes in proinflammatory cytokines, caspase-1, IL-1β and TNF-α, and M1/M2 macrophage polarization. Increased AMPK/Sirt1 expression and decreased NLRP3 expression brought about by administration of the antagomir, reduced downstream proinflammatory cytokines. Flow cytometry analysis showed the M1 to M2 conversion and inhibition of AMPK expression reduced the effect of miR-31-5p antagomir. We have planned further studies to improve our findings: (1) the sample size should be expanded for transcriptome sequencing to obtain more differentially expressed miRNAs; (2) flow cytometry analysis should be performed with macrophages from mouse colonic epithelial cells; (3) further mouse experiments should be performed to verify that the effect of the antagomir of miR-31-5p is suppressed when AMPK is inhibited; (4) macrophages should be stimulated with IL-4 *in vitro* to observe changes in M2 proteins with the miR-31-5p antagomir; (5) mouse primary bone marrow macrophages (BMDM) should be used to verify the accuracy and rationality of experiments *in vitro*. Nonetheless, the miR-31-5p antagomir consistently activated the AMPK signaling pathway to achieve therapeutic effects in colitis, restoring epithelial barrier function, downregulating inflammatory cytokines and promoting M1 to M2 macrophage transformation. The mechanism diagram is shown in Figure 9.

UC involves various types of cells, such as dendritic cells, B cells, and T cells. In order to demonstrate the specificity of the role of miR-31-5p/AMPK/Sirt1 in macrophages, future research will need to conduct similar studies in various cells within the intestinal tract of UC to elucidate the mechanism of this pathway.

## CONCLUSION

In summary, miR-31-5p antagomir ameliorated DSS-induced colitis in mice by transforming M1-type macrophages into M2-type macrophages, reducing inflammatory infiltration by macrophages and restoring intestinal epithelial barrier function. The AMPK/Sirt1/NLRP3 signaling pathway was likely to have been involved.
